# A proline derivative-enriched methanol fraction from *Sideroxylon obtusifolium* leaves (MFSOL) stimulates human keratinocyte cells and exerts a healing effect in a burn wound model

**DOI:** 10.1590/1414-431X2021e10700

**Published:** 2021-05-31

**Authors:** T.F.G. Souza, T.M. Pierdoná, F.S. Macedo, P.E.A. Aquino, G.F.P. Rangel, R.S. Duarte, L.M.A. Silva, G.S.B. Viana, A.P.N.N. Alves, R.C. Montenegro, D.V. Wilke, E.R. Silveira, N.M.N. Alencar

**Affiliations:** 1 Departamento de Fisiologia e Farmacologia, Faculdade de Medicina, Universidade Federal do Ceará, Fortaleza, CE, Brasil; 2 Faculty of Kinesiology and Recreation Management, Children's Hospital Research Institute of Manitoba, University of Manitoba, Winnipeg, Manitoba, Canada; 3 Embrapa Agroindustria Tropical, Fortaleza, CE, Brasil; 4 Departamento de Clínica Odontológica, Faculdade de Farmácia, Odontologia e Enfermagem, Universidade Federal do Ceará, Fortaleza, CE, Brasil; 5 Departamento de Química Orgânica e Inorgânica, Centro de Ciências, Universidade Federal do Ceará, Fortaleza, CE, Brasil

**Keywords:** *Sideroxylon obtusifolium* (Roem. & Schult.), Methanol fraction, Human keratinocytes, Wound healing, Burn wounds

## Abstract

It was previously demonstrated that the methanol fraction of *Sideroxylon obtusifolium* (MFSOL) promoted anti-inflammatory and healing activity in excisional wounds. Thus, the present work investigated the healing effects of MFSOL on human keratinocyte cells (HaCaT) and experimental burn model injuries. HaCaT cells were used to study MFSOL's effect on cell migration and proliferation rates. Female Swiss mice were subjected to a second-degree superficial burn protocol and divided into four treatment groups: Vehicle, 1.0% silver sulfadiazine, and 0.5 or 1.0% MFSOL Cream (CrMFSOL). Samples were collected to quantify the inflammatory mediators, and histological analyses were performed after 3, 7, and 14 days. The results showed that MFSOL (50 μg/mL) stimulated HaCaT cells by increasing proliferation and migration rates. Moreover, 0.5% CrMFSOL attenuated myeloperoxidase (MPO) activity and also stimulated the release of interleukin (IL)-1β and IL-10 after 3 days of treatment. CrMFSOL (0.5%) also enhanced wound contraction, promoted improvement of tissue remodeling, and increased collagen production after 7 days and VEGF release after 14 days. Therefore, MFSOL stimulated human keratinocyte (HaCaT) cells and improved wound healing via modulation of inflammatory mediators of burn injuries.

## Introduction

The reconstruction of epidermal barrier rupture through re-epithelialization is essential to reduce wound infection risk and to recover normal skin function (1). Burn healing processes are affected by the interaction among cells and molecules, including growth factors, cytokines, and chemokines. Initially, the inflammatory response occurs to prevent the invasion of pathogens and to activate fundamental signals. It is followed by the proliferative phase, when fibroblasts and keratinocytes restore tissue damage through collagen synthesis, epithelialization, angiogenesis, and production of extracellular matrix (2,3). Infection by microorganisms causes a delay in the healing process, increased morbidity, and worse life quality. Thus, topical antibiotic treatment is a priority in burn patients. In this respect, 1% silver sulfadiazine is the gold standard (4).

The use of medicinal plants in Brazil is still common due to both the high costs of medicines and easy access to medicinal plants, which are widely sold in markets throughout the country (5). *Sideroxylon obtusifolium* (Roem. & Schult.) T.D. Penn is a native tree in Central and South America. It is common in the Brazilian semiarid region (Caatinga), where it is known as “quixaba” due to its round berries. It is widely used in folk medicine particularly in the cities of Northeast Brazil, where the dry parts of leaves or trunk bark are sold in local markets with claims of anti-inflammatory and healing properties (6).

The risk of extinction of the species by the predatory extraction of its bark has attracted attention of researchers to other plant parts, such as the leaves. Two studies with the hydroalcoholic fractions obtained from leaves of *S. obtusifolium* showed antifungal activity against *Candida albicans* (7,8). Another study demonstrated the antinociceptive and anti-inflammatory properties of an L-proline derivative, N-methyl-(2S,4R)-trans-4-hydroxy-L-proline (NMP), identified and isolated from the leaves of *S. obtusifolium* (9). NMP is present in the decoction from leaves of *S. obtusifolium*, and mainly in the methanol soluble fraction after methanol extraction. Also, NMP shows healing activity on excisional wounds related to its anti-inflammatory and antioxidant actions (10). However, it is unknown whether the proline derivative-enriched methanol fraction (MFSOL) is involved in the healing process of wounds caused by burns. We hypothesized that MFSOL would promote improvement in burn healing, possibly by stimulating migration and proliferation of keratinocytes. These effects are essential for the healing process of burns and other wounds that heal slowly, which present a high risk of infection and are difficult to treat. The results of this study demonstrated that MFSOL attenuated the inflammatory phase and stimulated the contraction of burns, corroborated by the *in vitro* stimulating migration effect of human keratinocytes.

## Material and Methods

### Plant material

Leaves of *S. obtusifolium* (Sapotaceae) were collected from specimens growing in the municipality of Mauriti, state of Ceará, Brazil, in August 2014. A voucher specimen (sample No. 10.648) was taxonomically validated by Dr. Maria Arlene Pessoa da Silva, at the “Herbario Caririense Dárdano de Andrade Lima”, of Regional University of Cariri, Ceará. The access to botanical material was registered in the Management System of Genetic Heritage and Associated Traditional Knowledge - SisGen, Brazil (Registration #AE9B987). The methanol soluble fraction of the *S. obtusifolium* leaf decoction (MFSOL) was obtained according to the procedure previously described to obtain a yellowish amorphous powder (9).

### Liquid chromatography-mass spectrometry analysis

Approximately 2 mg of MFSOL was solubilized in 2 mL of methanol. The supernatant was filtered through 0.2-μm PTFE membranes (Whatman, Merck, Germany), and 5 μL was injected into an Acquity UPLC (Waters Corp., USA) chromatographic system, coupled to a quadrupole/time of flight mass spectrometer (QTOF, Waters Corp.). The chromatographic separation was performed with an Acquity UPLC BEH column (150×2.1 mm, 1.7 μm; Waters Corp.) at a temperature of 40°C. The binary gradient elution system consisted of 0.1% formic acid in water (A) and 0.1% formic acid in acetonitrile (B), with linear gradient from 2 to 95% B (0-15 min), with a flow rate of 0.4 mL/min. The profiling was obtained by a Xevo Q-TOF mass spectrometer (Waters Corp.) with electrospray ionization (ESI) interface operating in negative ionization mode in the range of 110-1180 Da with scan time of 0.1 s. The desolvation gas was nitrogen at 350°C with flow rate of 500 L/h. The capillary and cone voltages were adjusted to 3 kV and 0.5 V, respectively. The mass accuracy and reproducibility were maintained by infusing lockmass (leucine-enkephalin, 0.2 ng/μL; [M-H]− ion at m/z 556.2771), and molecular formula assignments were obtained by the MassLynx 4.1 software (Waters Corp.).

In addition, the dataset was imported to the Mass Spectrometry - Data Independent AnaLysis software (MS-DIAL 3.82, RIKEN Center for Integrative Medical Sciences, Japan), to implement functions required for untargeted metabolomics, such as obtaining deconvoluted spectra, peak alignment, and filtering. Therefore, the unknown metabolites could be identified by their elemental formulas and *in silico* mass spectral fragmentation with MS-FINDER 3.22 (11). Structural elucidation and metabolite identification were performed considering the respective m/z values and fragmentation profile (MS/MS), with activated heuristic rules (12). Then, the compounds' putative identification was performed by comparing the data with information from the ChemSpider, KNApSAcK Core System, PubChem, Human Metabolite (HMDB), and Kyoto Encyclopedia of Genes and Genome (KEGG) databases. Finally, chemical identification was based on chemotaxonomy (family, genus, and species).

### qHNMR analysis

The quantitative ^1^H nuclear magnetic resonance (qHNMR) method was used to determine the *N*-methyl-(2S,4R)-*trans*-4-hydroxy-L-proline (NMP) concentration of MFSOL, where 10 g of MFSOL in 1 g aliquots was submitted to chromatography in an SPE C18 cartridge (20 g, Strata Phenomenex, USA) by elution with H_2_O (100 mL), H_2_O/MeOH 3:1 (60 mL), H_2_O/MeOH 1:1 (60 mL), and finally MeOH (60 mL). After lyophilization or rotary evaporation of the solvent, the fractions yielded 3.13, 3.03, 2.03, and 1.84 g, respectively. By ^1^H NMR, the presence of NMP was investigated and fractions were adsorbed on 20 g of silica gel for flash chromatography and then eluted with 10% CH_2_Cl_2_/MeOH (5 fractions of 50 mL), followed by 20% CH_2_Cl_2_/MeOH (9 fractions of 50 mL and 6 fractions of 100 mL) (13).

### Cell culture

Immortalized human keratinocyte cells (HaCaT, CLS Cell Lines Service) were provided by Dr. Raquel Carvalho Montenegro of the Pharmacogenetics Laboratory of the Drug Research and Development Center (NPDM), Federal University of Ceará. Cells were grown in Dulbecco's modified Eagle's medium (DMEM, Gibco®, USA), supplemented with 1.0% antibiotics (100 U/mL penicillin and 100 μg/mL streptomycin, Gibco®) and 10% fetal bovine serum (FBS, Gibco®), at 37°C in a 5% CO_2_ atmosphere in a humidified incubator. The cells were maintained in exponential growth through periodic maintenance.

### Cytotoxicity assays

The principle of the MTT assay is based on the mitochondrial cell viability, determined by reducing, through the succinate-tetrazole reductase enzyme system, the yellow tetrazolium salt (MTT) to a formazan salt, which is purple in color (14). After 24, 48, and 72 h of incubation with MFSOL, the cells received 20 μL of 3-(4,5-dimethylthiazolyl)-2,5-diphenyltetrazolium bromide (MTT) (5 mg/mL) and were incubated at 37°C for 3 h. Then, the medium was removed and 150 μL of dimethyl sulfoxide (DMSO) was added and the plate was homogenized for 5 min. The MFSOL was dissolved in sterile water and used to treat the cell culture at final concentrations of 6.25-100 μg/mL. The absorbance was determined by a microplate reader (Elisa Asys Expert Plus, UK) at a wavelength of 540 nm.

The sulphorhodamine B (SRB) assay was used to evaluate cell viability, based on the measurement of cellular protein content. HaCaT cells (2×10^4^ cells/mL) were plated onto 96-well plates and after 24, 48, or 72 h were treated with MFSOL (6.25-100 μg/mL). The treated cells were fixed with 10% trichloroacetic acid and incubated with SRB solution (0.4%) for 30 min. The excess dye was removed by washing repeatedly with 1% acetic acid. The protein-bound dye was dissolved in 10 mM Tris base solution and the absorbance was determined at 570 nm using a Microplate Autoreader (Multiskan FC, Thermo Scientific, Finland) (15). The results are reported as a percentage of cell viability.

### Scratch wound healing assay

The scratch wound healing assay was performed to determine the effects of MFSOL on the proliferation and migration of human keratinocytes. These cells were dispersed in DMEM with 10% FBS (5×10^4^ cells/mL) and incubated with 5% CO_2_ at 37°C in a 24-well plate. When the cells formed a confluent monolayer, they were scratched using a vertical tip with 200 μL on each well. Cell debris was removed and the wells were washed with phosphate buffered saline (PBS). Photomicrographs (200×) were taken after 24, 48, and 72 h of incubation with MFSOL (25, 50, and 100 μg/mL) in 1 mL of fresh medium (16). To evaluate only the migration, an antimitotic agent, mitomycin C (10 μg/mL; Sigma®, USA), was added for 1 h, following the scratch protocol. Immediately thereafter, the cells were treated with MFSOL (25 and 50 μg/mL) and, after obtaining photomicrographs, the migration was evaluated at the initial time and after incubation for 24 and 48 h (17). The open area of the scratch was measured using the TSCRATCH® software (ETH Zurich, CSE Lab, Switzerland) in each analysis period at the same initial site. The percentage of open area was measured using the formula: Open area (%) = open area at time *X* / open area at initial time × 100.

### Animals

Female Swiss mice (8 weeks old) were kept on a 12-h light/dark cycle, temperature of 23±2°C, and relative humidity of 55±10% at the Central Animal House of Federal University of Ceará (UFC). The animals received feed (Nuvilab, Quimtia®, Brazil) and water *ad libitum*. The experimental protocols were performed according to the ethical standards established in the Ethical Principles on Animal Experimentation adopted by Brazil's National Council for Control of Animal Experimentation (CONCEA), and were approved (No. 8862290518) by the Ethics Committee on the Use of Animals of Federal University of Ceará.

### Superficial burn model and treatment

The animals were anesthetized with xylazine hydrochloride (10 mg/kg, *ip*) and ketamine hydrochloride (100 mg/kg, *ip*). After anesthesia, the dorsal surface skin (4×2 cm) was shaved followed by asepsis with 1.0% iodopovidone and then 70% ethanol. A second-degree superficial burn was induced by direct contact with a heated square stainless steel plate (1 cm^2^) at 100°C for 6 s (18). The animals were divided into 4 groups: 1) control vehicle with Lanette cream base (anionic cream); 2) 0.5% CrMFSOL; 3) 1.0% CrMFSOL, in which the fraction was included in the dermatological cream base at the two concentrations; and 4) 1% silver sulfadiazine (Sulfa) with Lanette cream base as the positive control. Immediately after the injury, the treatments were administered once per day for 14 days. After induction of the lesion, the animals received 0.9% saline solution (*sc*) for fluid replacement and were kept in a warm environment under observation until complete recovery.

### Burn wound contraction

The lesions were measured and photographed on days 3, 5, 7, 9, 12, and 14 after induction of the superficial burn. A digital caliper was used to measure the horizontal (h) and vertical (v) size of the burn (mm), and then the area of the lesion was calculated by multiplying the two measurements, A = h × v (mm^2^). The rate of contraction of the lesion was calculated by the formula: Contraction (%) = (initial area - evaluation area) / (initial area) × 100 (19). After examination of the macroscopic wound healing (n=10/group), all mice were euthanized using an anesthetic overdose (xylazine and ketamine) and tissues were collected immediately and kept at -80°C for biochemical analyses.

### Tissue collection

The animals were euthanized with an overdose of xylazine hydrochloride (30 mg/kg, *ip*) and ketamine hydrochloride (300 mg/kg, *ip*) 3, 7, and 14 days after treatment. Burn wounds from each animal were removed as follows: half lesion on the 3rd day for determination of myeloperoxidase activity and another half to analyze mediators of inflammation; entire lesion on the 7th day for histology and collagen fiber analyses; and entire lesion on the 14th day for vascular endothelial growth factor (VEGF) measurement. Samples for biochemical evaluation were preserved in an ultra-freezer at -80°C and histological samples were immediately processed.

### Histological and collagen fibers analysis

The burn injuries were collected 7 days after injury for histological analysis. Samples were removed and fixed in 10% formol (pH 7.4) for 24 h (n=6/group). Tissues were submitted to dehydration and embedded in paraffin. Sections of those fragments were cut to 4-µm thick slices and stained with hematoxylin and eosin. Histopathological changes were evaluated by optical microscopy (Olympus BX 51, Japan). The histopathological parameters were determined and scored from 0 to 4, where 0 corresponded to: absence of ulcer (AU), remodeled connective tissue (RCT); score 1: AU, fibrosis (F), slight chronic inflammation (CI); score 2: presence of ulcer (PU), F, moderate CI; score 3: PU, chronic inflammation process (granulation tissue); and score 4: PU, acute process (dilated vessels, mixed inflammatory infiltrate with neutrophils) (20). Staining with picrosirius red (PSR) was performed to measure the collagen fibers in the connective tissue of the burn wounds. The Color Deconvolution (RGB) plugin of the ImageJ® software (NIH, USA) was used to measure the percentage of collagen area represented by the red image in relation to the total area of the image (21).

### Myeloperoxidase activity assay

The myeloperoxidase (MPO) activity was determined in burn biopsies 3 days after burn injury (22). The samples were homogenized using a Polytron tissue extractor (Ultra Stirrer, Brazil) in phosphate buffer pH 7.4, centrifuged at (13,953 *g*) for 30 min, and the pellet obtained was used to determine myeloperoxidase activity by oxidative reaction with 3,3′,5,5′-tetramethylbenzidine (TMB; 1.6 mM, Sigma®) and oxygen peroxide (H_2_O_2_; 0.5 mM). The number of neutrophils was quantified from a standard neutrophil curve (1×10^5^ neutrophils/well). The absorbance of the samples (n=6/group) was quantified in a Microplate Autoreader (Multiskan FC, Thermo Scientific) at a wavelength of 450 nm and the results are reported as the number of neutrophils/mg of tissue (cell/mg of tissue).

### Mediators of inflammation and vascular endothelial growth factor measurement

The biopsies of the burns were performed 3 days after injury to quantify the release of the inflammatory mediators tumor necrosis factor (TNF)-α, interleukin (IL)-1β, and IL-10, and after 14 days to quantity the growth factor VEGF (23). Tissues were triturated and homogenized at 10% (mg tissue/μL) at 4°C in PBS solution (pH 7.4), and the residues were removed after centrifugation at 6,976 *g* at 4°C for 5 min. The protocol was performed according to the manufacturer's recommendation for the conventional sandwich technique (R&D Systems®, USA ). After the ELISA protocol, the absorbance of the samples was quantified with a spectrophotometer at wavelength of 450 nm. The results are reported as cytokines/mL of homogenate and the concentration of the samples was obtained from a serial dilution of recombinant cytokine standard (n=6/group).

### Statistical analysis

Data are reported as mean ± SE of each experimental group. Statistical comparisons of the data were performed by one-way ANOVA followed by the Tukey post-test using GraphPad Prism version 5.0 (USA). Differences between groups were considered significant when P≤0.05.

## Results

### Identification of compounds in MFSOL of *S. obtusifolium* leaves

**Figure 1:**
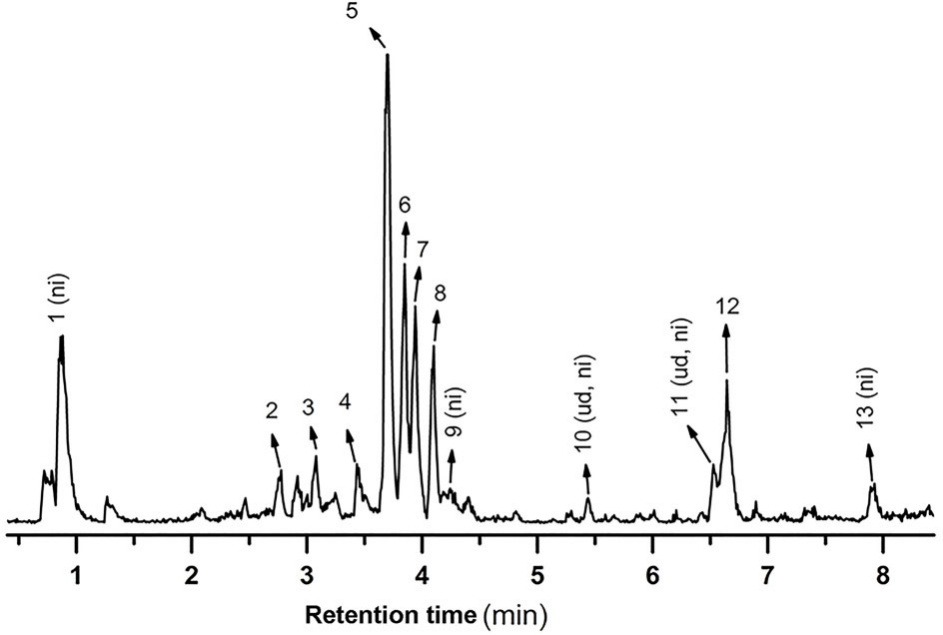
Typical base peak intensity (BPI) chromatogram of methanol fraction from leaf decoction of *Sideroxylon obtusifolium* (MFSOL) in the negative ionization mode. ni: not identified; ud: up to 1000 DA.

Some of the major compounds were identified according to their *m*/*z* and fragmentation profiles. Figure 1 shows the typical base peak intensity (BPI) chromatogram of the methanol fraction from the leaf decoction. Also, Table 1 shows the compounds with their retention times (rt), *m*/*z* in negative ionization mode, molecular formula, putative identification, and respective reference. ^1^H NMR was used to detect the presence of NMP just for the water fraction to yield 1.43 g of pure NMP (14.3%). This yield is in agreement with a previous finding by qNMR (13). The NMP spectrum extracted from the fraction corresponded to the pure isolated compound *N*-methyl-(2S,4R)-*trans*-4-hydroxy-L-proline (Supplementary Figures S1 and S2).

**Table 1:** Putative identification of the secondary metabolites present in the methanol fraction from leaf decoction of *Sideroxylon obtusifolium*.

Peak	R_t_ (min)	Negative ionization mode			Molecular formula	Putative identification	References
		MS [M-H]^-^	MS/MS	ppm			
**1**	0.89	317.0504	267.0718, 191.0534	-1.6	C_12_H_14_O_10_	ni	-
**2**	2.77	577.1335	289.0699, 425.0914, 407.0716, 245.0608, 125.0250	-1.9	C_30_H_26_O_12_	Procyanidin	-
**3**	3.06	289.0714	245.0822, 125.0243	0.7	C_15_H_14_O_6_	Catechin/epicatechin	-
**4**	3.45	865.2018	771.1993, 577.1306, 289.0768, 245.0540	4.4	C_45_H_38_O_18_	Procyanidin C1/Proanthocyanidin trimer	-
**5**	3.70	755.2028	625.1404, 300.0265	-0.9	C_33_H_40_O_20_	Kaempfero-3-O-(O-α-L-rhamnopyranosyl-(1→2)-O-[β-D-glucopyranosyl-(1→3)]-β-D-galactopyranoside) isomer I	(6)
**6**	3.85	771.1991	300.0289	1.4	C_33_H_40_O_21_	Quercetin-3-O-(O-α-L-rhamnopyranosyl-(1→2)-O-[β-D-glucopyranosyl-(1→3)]-β-D-galactopyranoside) isomer I	(6)
**7**	3.93	771.2008	609.1458, 300.0260	3.1	C_33_H_40_O_21_	Quercetin-3-O-(O-α-L-rhamnopyranosyl-(1→2)-O-[β-D-gluco- pyranosyl-(1→3)]-β-D-galactopyranoside) isomer II	(6)
**8**	4.09	755.2042	609.1447	0.9	C_33_H_40_O_20_	Kaempferol-3-O-(O-α-L-rhamnopyranosyl-(1→2)-O-[β-D-glucopyranosyl-(1→3)]-β-D-galactopyranoside) isomer II	(6)
**9**	4.23	785.2330	755.1964, 593.1561, 463.0878, 300.0277	-0.5	C_45_H_38_O_13_	ni	-
**10**	5.43	Up to 1000	1121.5522, 643.2460, 583.2434			ud	-
**11**	6.52	Up to 1000	1075.5504			ud	-
**12**	6.64	1339 (Up to 1000)	1075.5284, 665.3899, 269.0430	-	C_62_H_100_O_31_	3-O-(β-D-glucopyranosyl)-protobassic acid 28-O-β-D-apiofuranosyl-(1→3)-O-[O-β-D-apiofuranosyl-(1→3)-β-D-xylopyranosyl-(1→4)]-O-α-L-rhamnopyranosyl-(1→2)-α-L-arabinopyranosyl ester	(6)
**13**	7.92	665.3960	325.1620, 116.9397	0.0	C_29_H_62_O_16_	ni	-

R_t_: retention time; ni: non-identified, ud: up to 1000 DA.

### MFSOL enhanced the viability of human keratinocyte HaCaT cells

**Figure 2:**
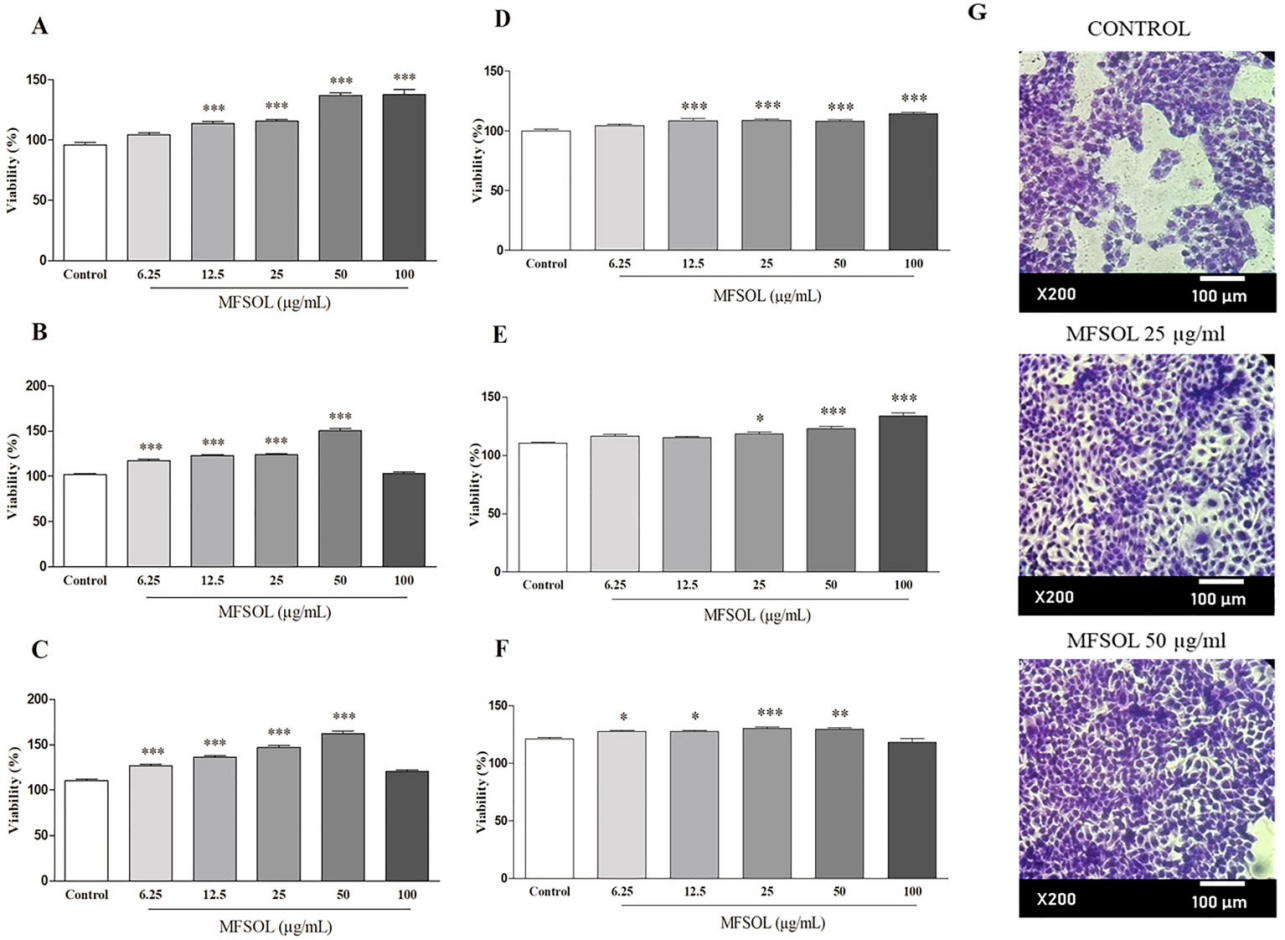
Effect of methanol fraction of *Sideroxylon obtusifolium* (MFSOL) on cell viability of human keratinocytes (HaCaT). Increased viability of keratinocytes was produced by MFSOL (6.25-100 μg/mL) after 24 (**A**), 48 (**B**), and 72 (**C**) h of incubation by the MTT assay and SRB assay (**D**, **E**, and **F**), respectively. To illustrate the HaCaT density, photographs were taken at 200× magnification (scale bar 100 μm) 48 h after treatment with 25 and 50 μg/mL of MFSOL (**G**). Data are reported as means±SE of at least three independent experiments. *P<0.05, **P<0.01, ***P<0.001, compared to the Control (Vehicle) group (one-way ANOVA followed by the Tukey post-test).

The proliferative assay revealed that MFSOL was not cytotoxic to human keratinocytes at any of the incubation periods. MFSOL was able to increase mitochondrial viability (by the MTT assay) after 24, 48, and 72 h at concentrations of 12.5-50 μg/mL (P<0.001). The concentration of 50 μg/mL of MFSOL stimulated increases in cell viability of 42.8% (after 24 h), 47.5% (after 48 h), and 46.8% (after 72 h) compared to the Control group (P<0.001) (Figure 2A, B, and C, respectively). At the highest concentration (100 μg/mL), the increase was 43.6% (P<0.001) after 24 h. The SRB assay also revealed an increase of viable cells, but less pronounced than indicated by the MTT assay. After 24 and 48 h, the concentration of 100 μg/mL of MFSOL increased the viability of HaCaT cells by 14.5 and 21% (P<0.001), which was also observed with 50 μg/mL (8.2 and 11.21%, respectively; P<0.001). These effects were less pronounced after 72 h, when 25 and 50 μg/mL of MFSOL caused viability increases of 7.1 and 7.4% (P<0.001).

### MFSOL stimulated proliferation and migration of keratinocytes in the HaCaT scratch assay

**Figure 3:**
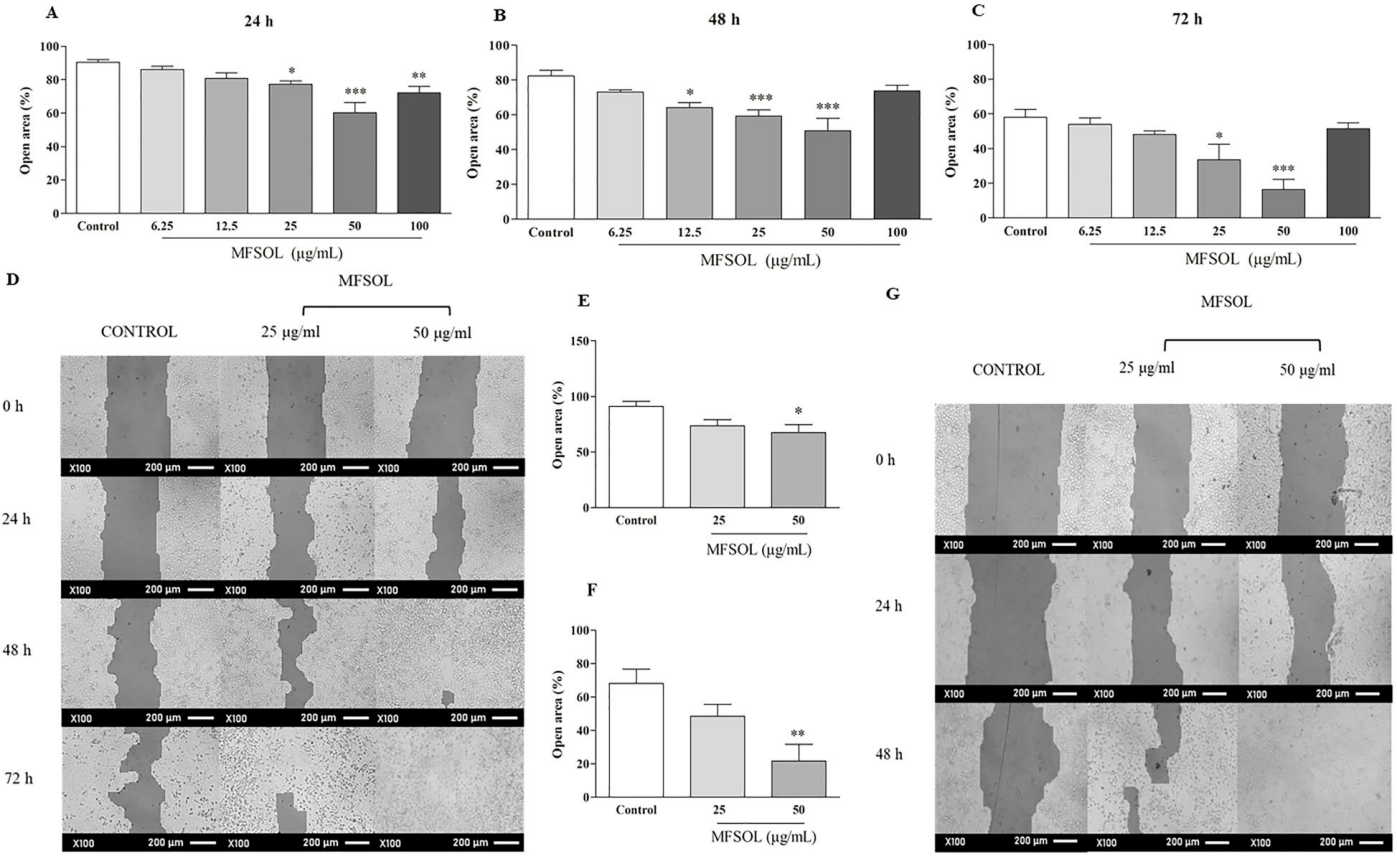
Methanol fraction of *Sideroxylon obtusifolium* (MFSOL) stimulated cell proliferation and migration rates of HaCaT in the scratch assay. A scratch was produced in a monolayer of HaCaT cells and photographs were taken, before (0 h) and 24 (**A**), 48 (**B**), and 72 h (**C**) after treatment with MFSOL (100× magnification; scale bar 200 μm) (**D**). Pretreatment with mitomycin C (10 μg/mL) was also performed one hour before the introduction of the scratch to inhibit cell proliferation, allowing only evaluation of cell migration after 24 h (**E**) and 48 h (**F**). The wells were photographed (100×, scale bar 200 μm) after 24 and 48 h (**G**). The data are reported as means±SE. *P<0.05, **P<0.01, ***P<0.001 compared to the Control group (water) (one-way ANOVA followed by the Tukey test).

To evaluate the effect of MFSOL on cell proliferation and migration of HaCaT, the *in vitro* healing test known as the scratch assay was used. Based on the results of the MTT and SRB assays, we selected concentrations of 25, 50, and 100 μg/mL to perform the scratch assay. After 24 h, the percentages of open area observed after treatment with 25 μg/mL (P<0.05) and 50 μg/mL (P<0.001) of MFSOL were reduced by 14.5 and 34%, respectively, compared to the Control (Figure 3A). This effect was also observed 48 h after treatment with 12.5 μg/mL (22%; P<0.05), 25 μg/mL (28%; P<0.001), and 50 μg/mL (38%; P<0.001) of MFSOL, respectively, compared to the Control (Figure 3B). Finally, after 72 h, the open area was reduced by treatment with 25 μg/mL (42.1%; P<0.05) and 50 μg/mL (72.6%) of MFSOL (P<0.001) compared to the Control (Figure 3C). The effect of MFSOL on HaCaT was photographed and visualized through rapid panotype staining. This was observed by the higher cell density after treatment with 25 and 50 μg/mL of MFSOL compared with the Control (Figure 3D). Considering the stimulatory effect of MFSOL on cell proliferation and migration, we used mitomycin C to inhibit cell duplication and thus enable the isolated evaluation of the effect of MFSOL on cell migration. At the concentration of 50 μg/mL, it was efficient in stimulating the migration of the cells to the center of the open area by 26% after 24 h (P<0.05) and 67% after 48 h (P<0.01) of incubation compared to the Control (Figure 3E, F, and G).

### CrMFSOL reduced the area of superficial burns

**Figure 4:**
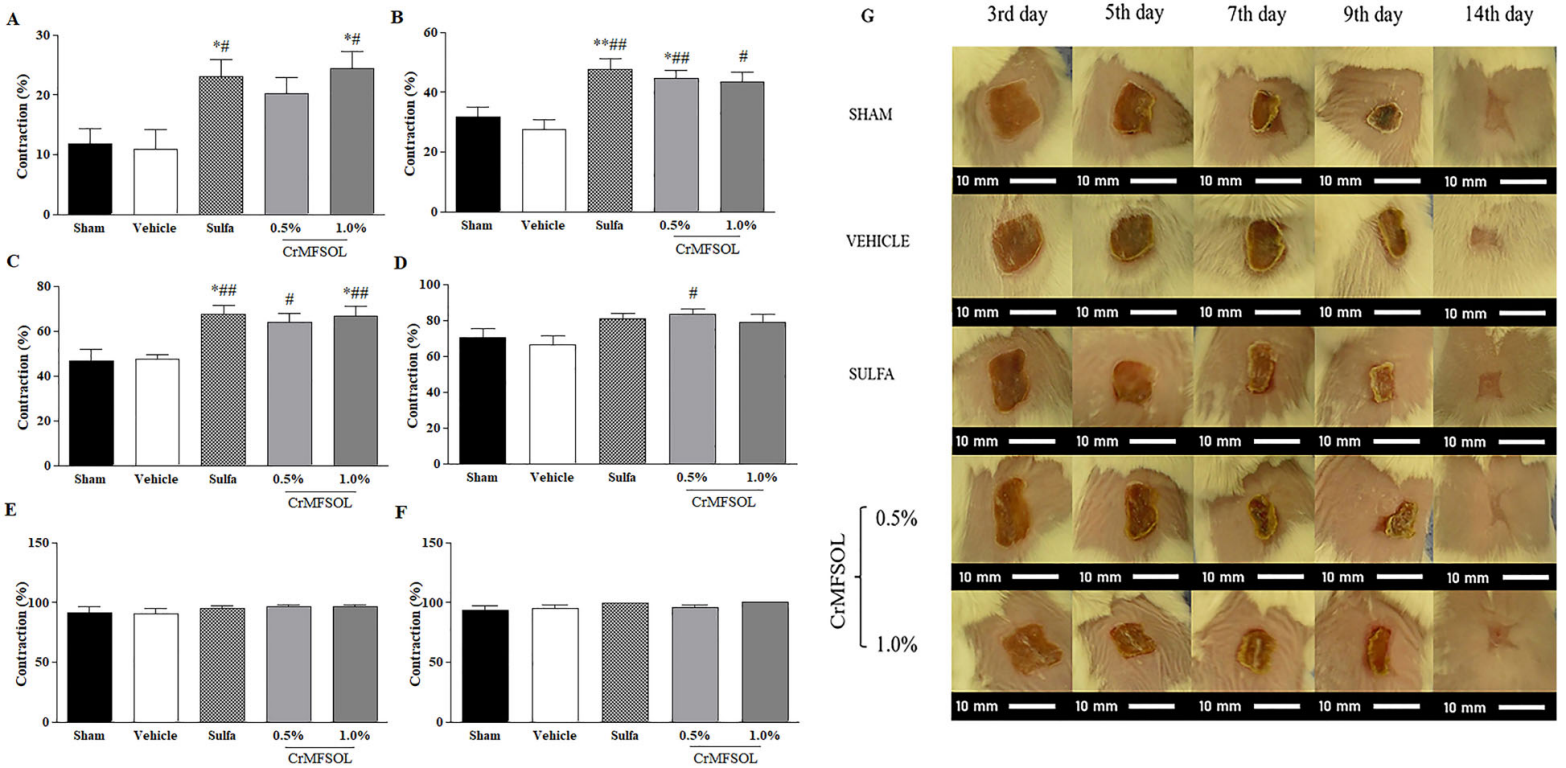
Effect of methanol fraction of *Sideroxylon obtusifolium* (MFSOL) on the contraction of induced burns in mice. The percentage of contraction of the burns was measured after 3, 5, 7, 9, 12, and 14 days (**A**, **B**, **C**, **D**, **E,** and **F**, respectively). The animals were treated with a single daily application of dermatological creams (Cr) for 14 days. The CrMFSOL group received 0.5 and 1.0% MFSOL, the Sulfa group received 1% silver sulfadiazine cream, and the Vehicle group was exposed to the base vehicle cream without any addition of active substance. The superficial burns were photographed for macroscopic monitoring on days 3, 5, 7, 9, 12, and 14 after the burn was produced (scale bar 10 mm) (**G**). One animal per group was chosen to represent the group according to the lesion contraction results. The data are reported as mean±SE. *P<0.05, **P<0.01 compared to the Sham group; ^#^P<0.05, ^##^P<0.01 compared to the Vehicle group, (n=10 animals/group) (one-way ANOVA followed by the Tukey test).

In the superficial burn model, the contraction of lesions was measured for 14 days to monitor the evolution of the area of superficial burns. The contraction rate of the Sham and Vehicle groups was similar in all the periods studied (Figure 4A-F). After 3 days, the treatment with 1.0% CrMFSOL caused an increase of 123.2% (P<0.05) in the contraction of lesions, while, the Sulfa group (P<0.05) presented a similar effect (110.7%) compared to the Vehicle group (Figure 4A). After 5 days (Figure 4B) and 7 days (Figure 4C), the groups receiving 0.5% CrMFSOL (P<0.01; P<0.05), 1.0% CrMFSOL (P<0.05; P<0.01), and Sulfa (P<0.01; P<0.01) showed increases of lesion contraction, respectively, compared to the Vehicle group (Figure 4C). After 9 days, only the group receiving 0.5% CrMFSOL had greater (26%) lesion contraction (P<0.05) compared to the Vehicle group (Figure 4D). Near the closure of the lesion, after 12 and 14 days of analysis, none of the groups presented differences (Figure 4E and F). The photographs depict the evolution during the healing process on the 3rd, 5th, 7th, 9th, 12th, and 14th days (Figure 4G).

### CrMFSOL promoted tissue remodeling improvement, re-epithelialization, and collagen deposition of superficial burns

**Figure 5:**
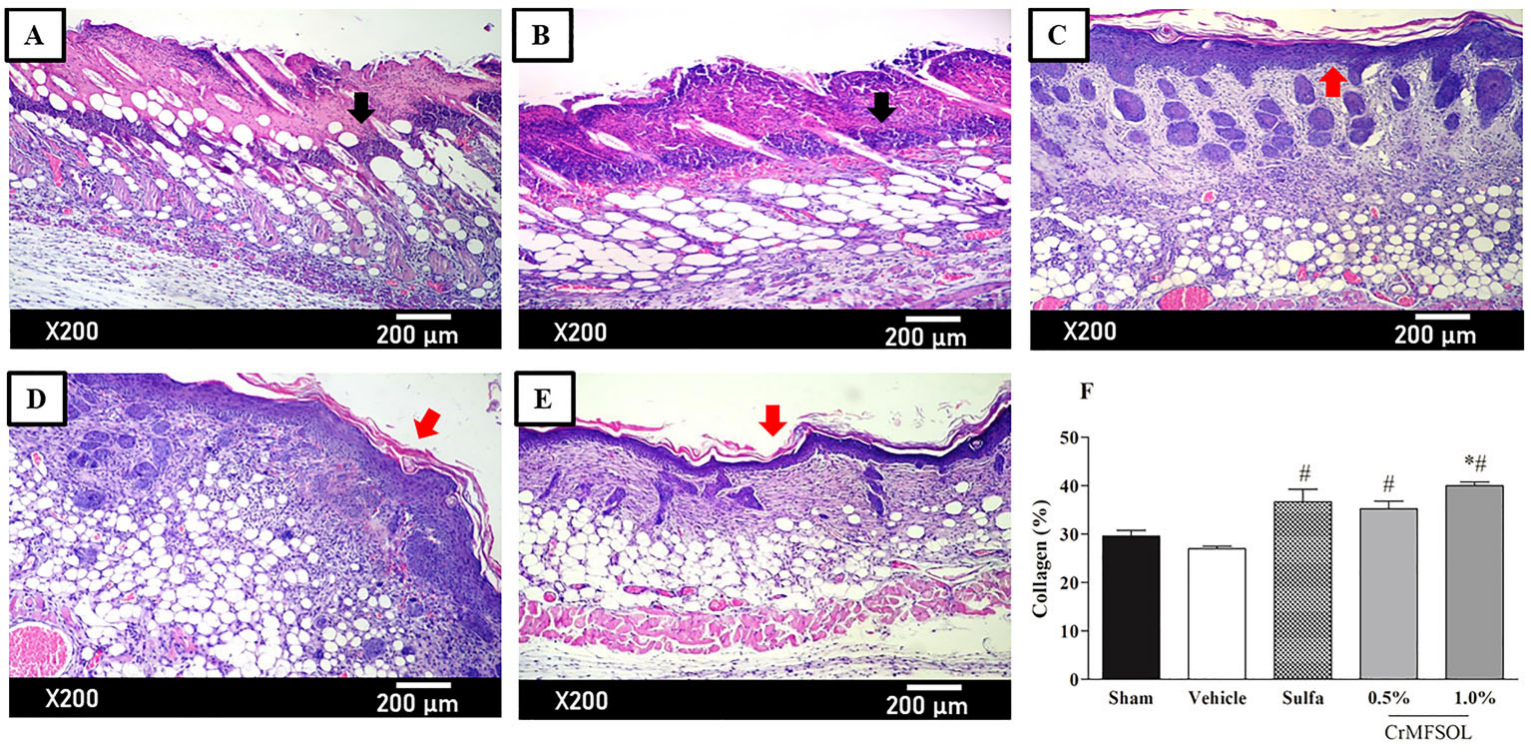
Cream of methanol fraction of *Sideroxylon obtusifolium* (CrMFSOL) promoted improved tissue remodeling, reepithelialization, and stimulated collagen production on superficial burns 7 days after treatment. Photomicrographs of histological slides (hematoxylin-eosin staining) of burn lesions on the 7th day after injury for the groups Sham (**A**), Vehicle (**B**), silver sulfadiazine (**C**), 0.5% CrMFSOL (**D**), and 1.0% CrMFSOL (**E**) (n=6 animals/group). Black arrows indicate the presence of acute inflammatory infiltrate in groups **A** and **B**, while red arrows indicate the presence of atrophic epithelium, presence of a corneal layer, and absence of ulcer in groups **C**, **D**, and **E**. The lesions were stained with picrosirius red and photographed in six fields (200×, scale bar 200 μm). The percentage of total collagen present on the 7th day was determined by the ImageJ® software (**F**). The data are reported as mean±SE of the six areas of each wound per group. *P<0.05 compared to the Sham group; ^#^P<0.05 compared to the Vehicle group (n=6 animals/group per day) (one-way ANOVA followed by the Tukey test).

Histopathological analysis of samples of superficial burns in mice showed that after 7 days the Sham and Vehicle groups had ulcers, granulation tissue, and the presence of acute inflammatory infiltrate (Figure 5A and B). On the other hand, in the Sulfa and the 0.5 and 1.0% CrMFSOL groups (P<0.05), a thin epithelium covering the ulcer was present, along with a corneal layer protecting the newly formed epithelium. It was still possible to observe the transition from acute to chronic inflammatory infiltrate and the remodeling of connective tissue (Figure 5C, D, and E, respectively). To determine the total collagen deposition in the tissue, picrosirius red staining was used 7 after days of treatment. The Vehicle and Sham groups had similar percentages of total collagen. The corresponding percentages of collagen production in the Sulfa and the 0.5 and 1.0% CrMFSOL groups were 36, 30.7, and 48.1% (P<0.05), respectively compared with the Vehicle group (Figure 5F).

### Effect of CrMFSOL on cytokine inflammatory status of superficial burns

**Figure 6:**
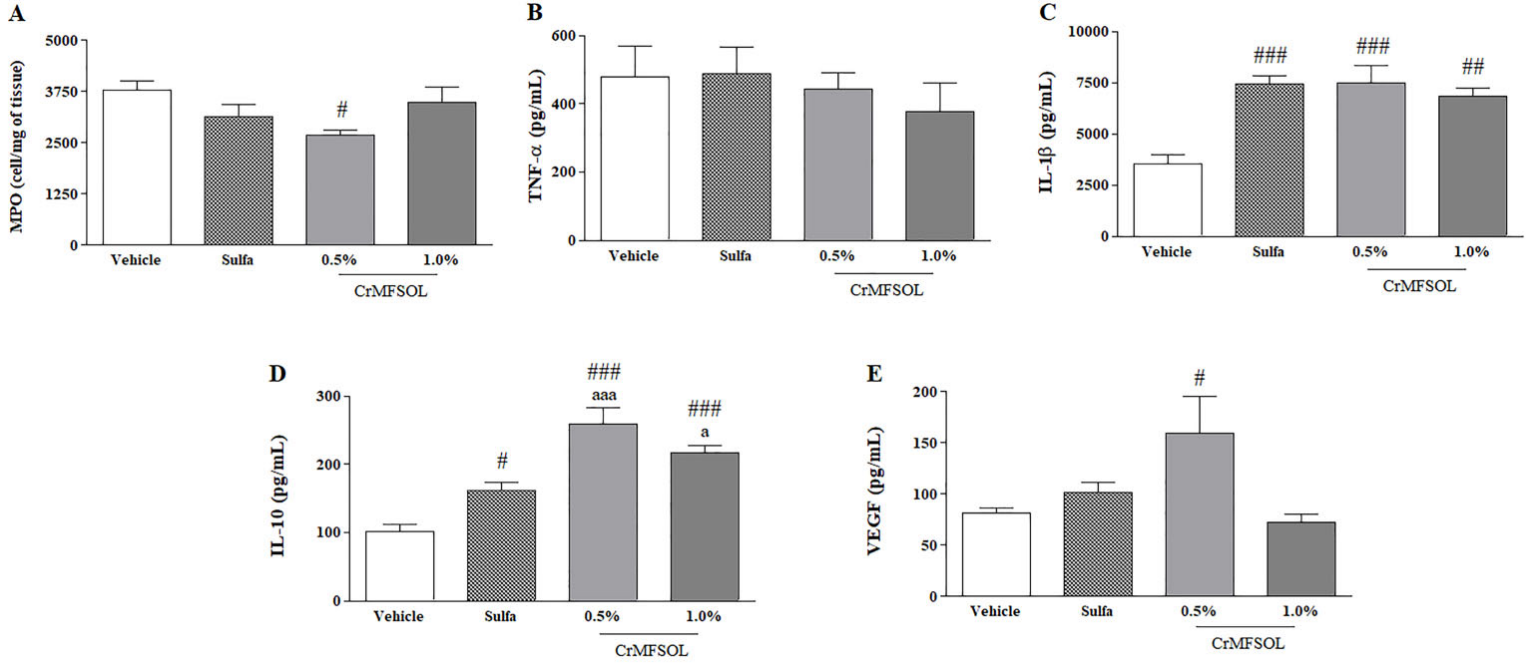
The treatment with methanol fraction of *Sideroxylon obtusifolium* cream (CrMFSOL) reduced myeloperoxidase activity (MPO) and stimulated release of interleukin (IL)-1β and IL-10 after 3 days, and vascular endothelial growth factor (VEGF) after 14 days. The levels of tumor necrosis factor (TNF)-α were not reduced significantly. The data are reported as mean±SE. ^#^P<0.05, ^##^P<0.01, ^###^P<0.001 compared to the Vehicle group; ^a^P<0.05 and ^aaa^P<0.001 compared to the Sulfa group n=6 animals/group per day) (one-way ANOVA followed by the Tukey test).

Treatment with 0.5% CrMFSOL reduced (P<0.05) the activity of MPO by 32% compared to the Vehicle group 3 days after burn injury (Figure 6A). Moreover, the levels of TNF-α were not altered by 0.5 or 1.0% CrMFSOL (Figure 6B). However, CrMFSOL at 0.5% (P<0.001) and 1.0% (P<0.01) increased IL-1β release by 112.5 and 94.4%, respectively, compared to the Vehicle group, as did treatment with Sulfa (P<0.001) (Figure 6C). Additionally, the release of IL-10, an anti-inflammatory cytokine, was higher in all treated groups compared with the Vehicle group. Treatment with 0.5% CrMFSOL caused increases of 153.7% (P<0.001) and 59.8% (P<0.01) compared to the Vehicle and Sulfa groups, respectively (Figure 6D).

### CrMFSOL stimulated release of VEGF in superficial burns

To evaluate the angiogenesis process, the VEGF level was measured 14 days after induced superficial burn injury. The treatment with 0.5% CrMFSOL increased VEGF release by 95.6% (P<0.05) compared to the Vehicle group (Figure 6E).

## Discussion

The present work reports, for the first time, the healing activity on burn wounds of a methanol fraction from *Sideroxylon obtusifolium* leaves (MFSOL). *S. obtusifolium* is one of the most important native species of the Caatinga biome targeted for conservation and sustainable maintenance. The plant has been mentioned by local experts as among the five most important native species with respect to therapeutic value, being used to treat pain and injuries from blows. However, the use of medicinal plants in the Caatinga biome often involves the removal of the bark, a practice that can kill the plant (24). In contrast to the traditional use of stem bark, this work improves the chances of conservation by demonstrating the medicinal properties of the leaves as well.

Besides the interest in bioactive compounds, this plant’s fruit also has a high nutritional potential (25). A previously published detailed metabolic profile of the ethanol extract of *S. obtusifolium* leaves revealed saponins and flavonoids as the main constituents. The authors also identified a new glycoside triterpene and flavonol glycosides (quercetin-glycoside, kaempferol-glycoside), which were also identified in MFSOL (6). In addition, catechin/epicatechin, procyanidin C1, and proanthocyanidin were identified in MFSOL. The use of natural products with anti-inflammatory, antioxidant, antibacterial, and pro-collagen synthesis properties can aid in wound healing. Bioactive constituents of the chemical classes of alkaloids, flavonoids, tannins, terpenoids, saponins, and phenolic compounds have been found to have healing benefits. These substances are generally absorbed well by the skin and modulate different stages of healing (26). Fujishima et al. identified 13 compounds in a hydroethanolic extract from the leaves of *C. americana*, mainly quercetin, kaempferol, glucoside derivatives, catechin, and epicatechin, which could explain the excellent healing effect (27).

MFSOL was not cytotoxic and promoted increased viability of human keratinocytes (HaCaT), suggesting an effect on the proliferation of HaCaT. In the scratch assay, MFSOL showed a proliferative and migratory effect on keratinocytes during wound healing. To evaluate only cell migration, mitomycin C, an antimitotic agent, was used to interfere with cell duplication in the scratch assay. MFSOL (25 and 50 μg/mL) stimulated not only proliferation but also the migration of HaCaT. Epidermal cell turnover is fundamental for rapid wound healing, in which keratinocytes are stimulated to proliferate by regulating growth factors and intercellular contact (28).

As a pharmacological model, the induction of superficial burns in mice was chosen to evaluate the effect of a topical cream (CrMFSOL) containing MFSOL at concentrations of 0.5 and 1.0% for 14 days. These concentrations of MFSOL corresponded to the best concentrations tested *in vitro* (50 and 100 µg/mL) in keratinocytes. The faster evolution of the contraction area of burn wounds, caused by treatment with CrMFSOL after 3, 5, 7, and 9 days, reduced the risk of infections and accelerated the other stages of healing, such as proliferation and remodeling. Its effect was similar to that observed in the Control group (silver sulfadiazine), which received the standard treatment for the prevention of infections and the promotion of burn healing (29).

Collagen is a structural protein responsible for tissue repair and wound healing. Proline and its metabolite (hydroxyproline) are unique amino acids and constitute one-third of amino acids present in collagen proteins. Proline also protects tissues from free radicals and reactive oxygen species (ROS), which induce tissue damage due to their antioxidant potential (30). Proline is only considered essential for the body in situations of burns and other injuries. It plays a crucial role in collagen synthesis and wound healing, mainly by regulating cell differentiation, cell nutrition, growth signaling factors, protein synthesis, and excretion of oxidants from the body (31). The high demand for proline during wound repair can cause a local proline deficiency. It has been suggested that the increase in proline bioavailability would be an interesting strategy to optimize collagen biosynthesis (32). According to Thangavel et al. (30), the topical treatment with CrMFSOL stimulated the early formation of new epithelium and remodeling of connective tissue, with a higher collagen deposition after 7 days. The incorporation of L-proline in a hydrogel accelerated wound healing by enhanced wound tissue regeneration and repair.

The progression of thermal injury damages adjacent capillaries, causing ischemia, which activates adhesion of polymorphonuclear cells such as neutrophils, which release inflammatory mediators and promote the production of ROS (33). While TNF-α release induces the fundamental inflammatory response in damaged tissue, the high level of IL-1β in mouse burns was correlated with the increased activity of epidermal keratinocytes. These cells work to restore the epidermal barrier function when stimulated by nanofibers of peptide hydrogels (34). The topical application of quercetin on full-thick epidermal burn wounds in rats produced a healing effect. It has been suggested that quercetin inhibits free radical damage of fibroblasts and keratinocytes, and this reduces inflammation and histamine release in burned skin and the surrounding tissue (35). Treatment with 0.5% CrMFSOL caused an increase in IL-1β levels, but it did not influence TNF-α release in burn injuries. Moreover, 0.5% CrMFSOL reduced MPO activity 3 days after treatment, which may have influenced the transition from acute to chronic inflammatory infiltrate after 7 days, earlier than the treatment with silver sulfadiazine. The ethanol extract of the inner bark of quixaba shows antinociceptive, analgesic, and anti-inflammatory effects, blocking leukocyte migration and establishing antioxidant activity, attributed to flavonoids (36). A polyphenol-enriched fraction from *Annona crassiflora* fruit peel containing epi-catechin, procyanidins B2 and C1, quercetin-glucoside, and kaempferol shows anti-inflammatory properties by reducing the activity of neutrophils and macrophages in cutaneous wounds. These compounds also favored the synthesis of collagen and the closure of wounds (37).

As an anti-inflammatory factor, IL-10 is also produced by epidermal keratinocytes and is important in the suppression of ROS and nitric oxide (NO) and the control of proinflammatory cytokines secreted by macrophages (38). Briefly, 0.5% CrMFSOL caused an increase in IL-10 level, while MFSOL was able to modulate the inflammatory response and contribute to healing of burned tissue. Furthermore, IL-1β has been reported to stimulate VEGF expression during inflammation, which consequently stimulates migration and proliferation of endothelial cells by the promotion of angiogenesis (39). One study reported that 0.3% quercetin treatment of wounds accelerates healing by a variety of biological effects: rapid wound contraction, modulation of inflammatory and anti-inflammatory cytokines, increased VEGF and TGF-b1, increased antioxidant status, and enhanced fibroblast proliferation, with better collagen deposition (40). Similarly, 0.5% CrMFSOL promoted a greater release of VEGF 14 days after treatment of burn injuries, which may have contributed to better epithelization and reorganization of the connective tissue.

### Conclusions

The methanol fraction extracted from the leaf decoction of *Sideroxylon obtusifolium* (MFSOL) demonstrated wound healing potential by stimulating proliferation and migration of human keratinocytes. Also, MFSOL modulated the inflammatory response and improved tissue repair of superficial burns in mice. Thus, the anti-inflammatory and wound healing potential of MFSOL provided support for further studies to enable the development of an herbal product for the treatment of burns and other wounds.

## Supplementary Material

Click here to view [pdf].
